# High-resolution ultrasound biomicroscopy for monitoring ovarian structures in mice

**DOI:** 10.1186/1477-7827-7-69

**Published:** 2009-07-06

**Authors:** Rajesh S Jaiswal, Jaswant Singh, Gregg P Adams

**Affiliations:** 1 Department of Veterinary Biomedical Sciences, Western College of Veterinary Medicine, University of Saskatchewan, Saskatoon, SK, S7N 5B4, Canada; 2 Alberta School of Business, University of Alberta, Edmonton, AB, Canada

## Abstract

**Background:**

Until recently, the limit of spatial resolution of ultrasound systems has prevented characterization of structures <1 mm. Hence, the study of ovarian follicular development in rodents has been based on one-time histological examination of excised tissues; i.e., longitudinal study of day-to-day ovarian changes has not been possible in mice and rats. The objective was to establish an ultrasonographic approach to study follicular and luteal dynamics in mice and rats.

**Methods:**

Experiment 1 was a pilot study to develop methods of immobilization (physical restraint vs. general anesthesia) and determine technical factors affecting ovarian images using ultrasound bio-microscopy in rats vs. mice. The hair coat was removed over the thoraco-lumber area using depilation cream, and a highly viscous acoustic gel was applied while the animals were maintained in sternal recumbency. In Experiment 2, changes in ovarian structures during the estrous cycle were monitored by twice daily ultrasonography in 10 mice for 2 estrous cycles.

**Results:**

Ovarian images were not distinct in rats due to attenuation of ultrasound waves. Physical restraint, without general anesthesia, was insufficient for immobilization in mice. By placing the transducer face over the dorsal flank, the kidney was visualized initially as a point of reference. A routine of moving the transducer a few millimetres caudo-laterally from the kidney was established to quickly and consistently localize the ovaries; the total time to scan both ovaries in a mouse was about 10 minutes. By comparing vaginal cytology with non-anesthetized controls, repeated exposure to anesthesia did not affect the estrous cycle. Temporal changes in the number of follicles in 3 different size categories support the hypothesis that follicles ≥ 20 microns develop in a wave-like fashion.

**Conclusion:**

The mouse is a suitable model for the study of ovarian dynamics using transcutaneous ultrasound bio-microscopy. Repeated general anesthesia for examination had no apparent effect on the estrous cycle, and preliminary results revealed a wave-like pattern of ovarian follicle development in mice.

## Background

Serial *in vivo *examination of the ovaries, through the use of ultrasonic imaging, has permitted detailed characterization of ovarian function in many species, and has led to a deeper understanding of endogenous mechanisms controlling follicular and luteal dynamics [1]. Using the bovine model as a basis [2], a wave-like pattern of development of ovarian antral follicle ≥4 mm has been documented in most species studied to date, e.g., ruminants [1], horses [3], camelids [4] wild species [5], humans [6], etc., using daily ultrasonographic monitoring of the size, number and relative location of follicles within each pair of ovaries. A wave-like developmental pattern refers to the synchronous growth of a group of follicles in response to a surge in circulating concentrations of follicle-stimulating hormone, usually followed by continued growth of a dominant follicle and rapid die-off of the subordinates [1]. More detailed study in cattle, using a higher resolution ultrasound system, has revealed that the wave-like pattern extends to antral follicles as small as 1 mm, and that the largest of the group at wave emergence is destined to become the dominant follicle [7]. However, the limit of spatial resolution of ultrasound systems has prevented characterization of the developmental pattern of follicles <1 mm. Hence, the study of follicular development in rodents has been based on one-time histological examination of excised tissues [8,9]; longitudinal study of day-to-day ovarian changes has not been possible in mice and rats.

The recent availability of a high-resolution ultrasound system (ultrasound bio-microscope) with high-frequency transducers (≥40 MHz) has enabled the imaging of structures as small as 70 μ [10], which in the ovaries includes primary, secondary, and small antral-stage follicles [11,12]. A limitation of such high-resolution ultrasonography, however, is rapid attenuation of the high-frequency sound waves and shallow tissue penetration (i.e., 7 to 10 mm) [13,14]. A rodent model was considered appropriate for initial attempts at high-resolution ultrasonography of ovarian structures because of its small size and thin tissues. In addition, rodents are relatively facile, abundant and inexpensive, and replication of ovarian studies involving a rodent model is faster due to a shorter estrous cycle of 4–5 days [15] compared to cattle (over 20 days) [1]. The findings about small follicle dynamics in a rodent model may also be extrapolative for cattle since morphology and developmental rate of follicles <1 mm is similar between the two species. In both rodents and cattle, primary follicles are 30–80 μm in diameter, secondary follicles are 80 to 250 μm, antrum formation (tertiary follicles) occurs at >250 μm [11,12,16], and the period required for the multiplication of granulosa cells (marker of follicular growth) called the 'doubling-time' is similar between rodents and cattle [17].

With the goal of developing an ultrasonographic approach to study the day-to-day dynamics of small follicle development, the specific objectives of the present study were to 1) determine if serial ovarian images could be obtained consistently in mice vs. rats using physical restraint vs. general anesthesia (Experiment 1), and 2) determine the technical feasibility of repeated ultrasound bio-microscopy to characterize the developmental dynamics of follicles in mice (Experiment 2). Development of such a technique will provide the opportunity for subsequent validation as a tool for characterizing follicle dynamics in rodents by comparison with histological assessment of follicle sizes and numbers [18].

## Methods

### Experiment 1

Adult CD-1 type female mice (n = 3) and Wistar type rats (n = 3) were used to compare methods of immobilization (physical restraint vs. anesthesia), and the quality of ovarian images obtained by ultrasound bio-microscopy in rats vs. mice. The hair coat over and around the thoraco-lumbar vertebrae was removed by hair-removal jelly (Nair^®^, Church and Dwight Company Inc., US). Physical restraint to limit movement without the need for general anesthesia was attempted with the use of the DecapiCone™ device (Braintree Scientific Inc., Braintree, MA, USA), a clear-plastic 50 mL test-tube, and a clear-plastic syringe case. For general anesthesia, an anesthetic induction box with 3.5% isoflurane (Abbott Laboratories Ltd., Saint-Laurent, QC, Canada) in oxygen gas was used, and the plane of anesthesia was regulated using a precision isoflurane vaporizer (Ohmeda, BOC Health Care, England). Anesthetized animals were placed in sternal recumbency on a thermo-regulated hot water blanket (42°C), and were maintained on 1.9% isoflurane during ultrasonographic examination.

**Figure 1:**
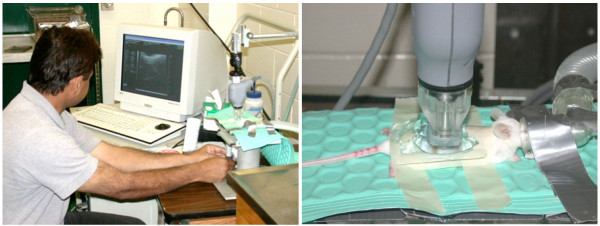
**Ultrasound imaging of the ovaries of anesthetized mice by transcutaneous ultrasound bio-microscopy using a 40 MHz transducer**.

Ovarian images were obtained using a high-resolution ultrasound bio-microscope (UBM; Vevo 660, Visual Sonics Inc., Toronto, Ontario, Canada) and a 40 MHz oscillating sector-array transducer. The transducer has a measured lateral resolution of 90 μm, axial resolution of 30 μm, and the depth of penetration of 7 to 10 mm, and it scans over an 8 mm linear path [13,14]. The transducer was placed in a stationary holder and could be moved along the vertical axis. The animal was placed on an examination stage and moved through two horizontal axes (forward-to-back and side-to-side) using a manually operated joy-stick. Acoustic gel was applied to the dorsal aspect of the thoraco-lumbar region; 3 acoustic ultrasound gels were tested: Eco Gel 200™ (Eco-Med Pharmaceutical Inc., Mississauga, ON, Canada), Aquasonic ultrasound transmission gel (Parker Laboratories Inc., Fairfield, NJ), and Triad™ (H & P industries Inc., Triad Disposables Inc., Mukwonago, WI). The transducer was lowered into the acoustic gel on the skin and the stage was moved until the ovary was visualized (Figure 1). Images were obtained while the stage was moved in 1 mm increments from the medial to the lateral aspect of the ovary. After examination, the acoustic gel was gently wiped-off and aloe vera gel (Alcohol free, Fruit of the Earth Inc, TX 76155) was applied to the skin to minimize irritation.

### Experiment 2

Adult CD-1 type mice (n = 20), 2.5 to 3 month of age, and weighing between 28 and 32 g were used. To ensure normal estrous cyclicity [15], female mice were housed in groups of 5 in a cage placed inside a larger cage in which a male mouse was allowed to dwell freely. Mice were kept under 14 h of light and 10 h of dark (i.e., lights on at 0600 h and out at 2000 h), at an ambient temperature of 21°C. Food and water were available *ad libitum*. Mice were allowed to acclimate for a period of 10 days before the start of the experiment, during which they were handled at least once a day to minimize the effect of handling stress on ovarian cyclicity during the experiment. The mice were assigned randomly to 2 groups (n = 10 per group) to be: 1) physically handled twice daily for two consecutive estrous cycles (controls), or 2) anesthetized and ultrasonographically scanned twice daily for a period similar to that of control mice.

Daily vaginal cytological examinations were conducted to monitor ovarian cyclicity of mice in both groups. The perineum was initially wiped clean with a saline-soaked cotton-tipped swab and about 25 μL of phosphate-buffered saline was gently dispensed and aspirated from vagina using micropipette (Eppendorf, Hamburg, Germany). The aspirate was examined by phase-contrast microscopy for nucleated epithelial cells, cornified epithelial cells, and leukocytes. The stage of the estrous cycle was estimated based on the predominant cell types [19].

Ovarian images for each scan were recorded digitally on a cineloop (300 frames/10 seconds cineloop). At the end of the study, cineloops were viewed frame-by-frame to prepare topographical sketches of follicles and to record their dimensions [7]. From the sketches, a count of follicles of different sizes was recorded [7]. Complete data (n = 10 per group) will be used elsewhere for reporting follicular developmental dynamics in the mouse. For the present study, n = 3 mice in the ultrasonographically scanned group and n = 10 mice in the control group were used to demonstrate the utility of ultrasound biomicroscopy for monitoring daily changes in ovarian structures.

Student's t-tests were used to make comparisons between control mice and those repeatedly scanned with regard to the inter-estrus interval (n = 10 per group), and the dimension of left vs. right ovaries (n = 3 repeatedly scanned mice). Data of the repeatedly scanned mice (n = 3) were centralized to the day of estrus. Follicles were divided into three size categories, 200 to 340 μ, 350 to 500 μ, and >500 μ to represent small antral, medium antral and large antral follicles [11,12]. Follicle number data were analyzed for the period between the morning of estrus to the evening of following proestrus (i.e., 12 h before next estrus) to determine the day, and follicle type effects [20]. If main effects or their interaction were statistically significant (*P *< 0.05), multiple comparisons were made using Tukey post hoc test. Data were analyzed by analysis of variance for repeated measures using the mixed procedure [21] in the Statistical Analysis System software package (SAS version 8.2 for MS Windows; SAS Institute Inc., Cary, NC). Five covariance structures (Compound Symmetry; Auto-Regressive 1; Unstructured; Unstructured 1; and Huynh-Feldt) were fitted to the data and the best model was selected based on the smallest Akaike information criterion values.

Procedures used in this study were reviewed and approved by the University of Saskatchewan Committee on Animal Care and Supply (protocol number 20040071), in accordance with the principles outlined by the Canadian Council on Animal Care.

## Results and Discussion

### Experiment 1

At the outset, physical restraint rather than anesthesia was a preferred method of immobilization to minimize examination-related effects on ovarian function. Female mice and rats are similar with regard to ovarian anatomy and the pattern of the estrous cycle [15,22]. In this regard, we expected that the rat might be a better rodent model for ultrasound examination based on its relatively larger size and greater docility than the mouse [15,22]. However, physical restraint without anesthesia was insufficient in both rats and mice as both remained restless during examinations, precluding acquisition of good-quality images.

The transducer face was usually positioned in the dorso-lateral flank region, just lateral to the epaxial muscles and just caudal to the last rib. Although a similar approach was used to locate the ovaries in rats and mice, image acquisition of the ovaries in rats was inconsistent and invariably of lower quality than that of mice, using a 40 MHz transducer. Difficulty in imaging the ovaries of rats was attributed to thicker skin and abdominal wall musculature in rats, which resulted in near-complete attenuation of the ultrasound beam. Hence, mice were chosen to establish the ultrasound bio-microscopy procedure for monitoring ovarian follicles on a repeated basis (Experiment 2). The following machine settings provided the most consistent images of ovarian structures in mice: 15 Hz frame rate, 10 mm field of view, 4 dB master gain, and 12.6 mm focal zone. A lower frequency transducer (e.g., 25 MHz) may provide better beam penetration and therefore be better suited for obtaining ovarian images in the rat.

Ovarian images were superior with the use of Eco Gel 200™ (Eco-Med Pharmaceutical Inc., Mississauga, ON, Canada) compared to other gels. Acoustic ultrasound gels are available in different viscosities, measured in centipoise (cps) units. Eco Gel 200™ is more viscous (80,000 to 100,000 cps) compared to the other acoustic gels used (35,000 to 70,000 cps). Gels with higher viscosity (e.g., Eco Gel 300TM, 110,000 to 120,000 cps) may provide greater conductivity for the present application, but have not yet been tested.

### Experiment 2

Repeated exposure to general anesthesia had no apparent effect on the estrous cycle of the mice. The inter-estrus interval (mean ± SEM) in scanned vs. control (non-anesthetized, non-examined) mice did not differ (4.8 ± 0.3 days vs. 4.9 ± 0.2 days, respectively, P = 0.77). The mouse ovary is small [~1 mm, 15], and was difficult to consistently localize until tissue landmarks were recognized. The ovaries were quickly located by first identifying the kidney as a major landmark, and then moving the transducer a few millimetres caudo-laterally from the caudal edge of the kidney. The time required to acquire images of both ovaries of a mouse was approximately 10 minutes.

It became apparent that application of hair removal gel at an interval of 3 days was insufficient to minimize the ultrasound-attenuating affect of the pelage. Consequently, depilation gel was applied daily or on alternate days. Ovarian images were further improved when a pool of acoustic gel was created on the dorsal body wall by making a masking-tape "dam" on the dorso-lateral walls of the abdomen (Figure 1).

**Figure 2:**
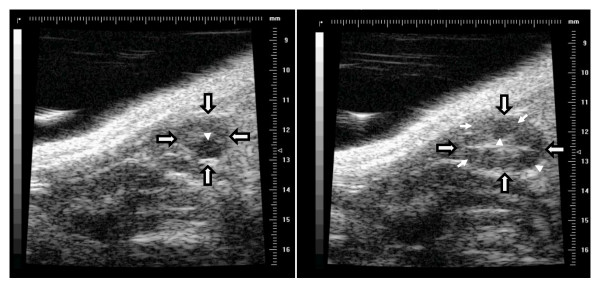
**Images of ovarian follicles and corpora lutea in mice using ultrasound biomicroscopy**. Arrows with a black outline define the external boundary of the ovary, white arrows point to corpora lutea, and arrow-heads indicate follicles. Numbers on the scale to the right of each image are in millimetres, with 0.1 mm increments.

The right and left ovaries were similar in size (2.45 mm ± 0.04 mm, P = 0.96). Ovarian follicles were recognized as roughly spherical, non-echogenic structures (Figure 2). There were between 19 and 41 antral follicles per pair of ovaries, ranging in diameter from 100 to 900 μm. Corpora lutea were detectable as slightly hypoechogenic spherical structures within the ovary, but were not easily discernable from the surrounding echogenic stromal tissue (Figure 2).

**Figure 3:**
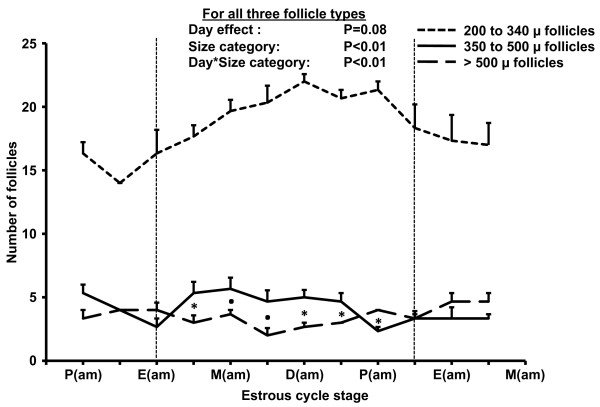
**Changes in the number (mean ± SEM) of small antral (200 to 340 μm), medium antral (350 to 500 μm), and large antral (>500 μm) follicles in mice (n = 3)**. Data were centralized to the day of estrus and analyzed between the morning of estrus and the evening of the following proestrus (i.e., 12 h before next estrus). Data from 24 h before and after the days of analysis are shown for completeness. P: Proestrus, E: Estrus, M: Metestrus, D: Diestrus. Asterisks (*) indicate differences between medium and large antral follicles (P < 0.01), whereas a dot (.) indicates a tendency for a difference (P = 0.06). The number of small antral follicles differed (P < 0.01) from the number of medium and large antral follicles on all days of the estrous cycle.

The number of small antral (200 to 340 μ), medium antral (350 to 500 μ), and large antral follicles (>500 μ) changed over days (Figure 3; day-by-category interaction *P *= 0.01), and the number of follicles in different size categories differed within days of the estrous cycle (P < 0.01). The day-by-category interaction was attributed to a successive rise and fall in the number of follicles in the small, medium and large categories, respectively, consistent with a wave-like pattern of follicles dynamics described in cattle [23]. The follicle number profile of medium antral (350 to 500 μ), and large antral follicles (>500 μ) during the estrous cycle is in agreement with early histologic studies in rats [9,11]. Histologic studies revealed that large follicles (400 to 500 μ) are "recruited" at estrus in response to a preovulatory surge gonadotropin surge. The recruited follicles grow during diestrus to a pre-ovulatory size (0.8–1.0 mm) and an average of 10.8 follicles ovulate in response to a surge in circulating concentrations of luteinizing hormone. Histologic data further suggest that only one wave of follicle development occurs during an interovulatory interval [9], consistent with the results of the present study.

**Figure 4:**
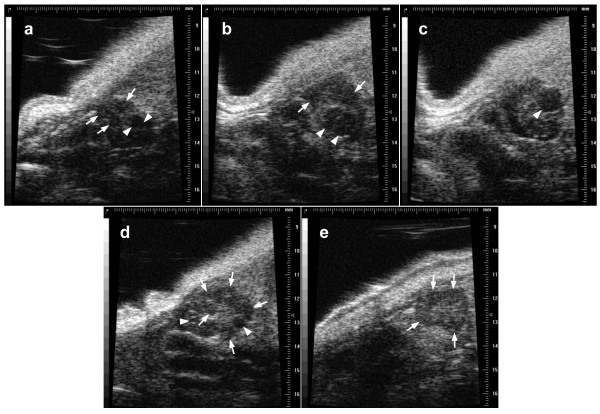
**Images of ovarian follicles and corpora lutea in mice using transcutaneous ultrasound biomicroscopy using a 40 MHz transducer**. Images were taken during proestrus (a), estrus (b, c), metestrus (d), and diestrus (e). White arrows indicate corpora lutea, and arrow heads indicate antral follicles.

The use of ultrasound bio-microscopy to monitor daily changes in mouse ovarian follicles was successful; however, the technique needs further refinement. The primary issue regarding interpretation of images was the difficulty in discerning corpora lutea from the surrounding ovarian stroma (Figure 4). A higher frequency transducer and the use of high-viscosity acoustic gel may help to identify corpora lutea. The image quality was also compromised in mice that developed skin rashes as a result of frequent depilation; however, the application of aloe vera cream was helpful in reducing pruritis. A validation study is needed to critically evaluate the power of ultrasound biomicroscopy for distinguishing follicles in different size categories and for identifying corpora lutea.

## Conclusion

The procedure for serial ultrasound biomicroscopy of murine ovaries *in vivo *was successful. The thicker skin of rats attenuated the high-frequency ultrasound waves; hence, in vivo rat ovaries were not amenable to imaging by transcutaneous ultrasound biomicroscopy using a 40 MHz transducer. General anesthesia was required to provide sufficient immobilization for consistent imaging of the ovaries on a repeated basis, but repeated anesthesia had no apparent effect on the estrous cycle. Ultrasonographic results of ovarian follicle dynamics in the present study conforms to early histological studies with regard to the follicular profile during the estrous cycle, and revealed a single wave of antral follicle development in mice.

## Competing interests

The authors declare that they have no competing interests.

## Authors' contributions

RJ was responsible for establishing the operating procedure of ultrasound biomicroscope for the monitoring of ovarian follicles, and for writing the initial draft of the manuscript. JS helped with technical details of the ultrasound equipment and for critical input and guidance. GPA supervised the study, provided critical input and guidance, and was responsible for manuscript revision and final submission. All authors have read and approved the final manuscript.
